# Fatal Eosinophilia-Associated Thrombotic Microangiopathy and the Role of Renal Charcot-Leyden Crystals

**DOI:** 10.1016/j.ekir.2025.05.015

**Published:** 2025-05-19

**Authors:** Shuhei Kurosawa, Keinosuke Hizuka, Yuna So, Yukihiro Yoshimura, Hiroko Minami, Takumi Idetsuka, Hiroyuki Hayashi, Rika Shimomura, Yoshikazu Yamaguchi, Hajime Hayami, Takashi Inoue, Yuko Kawamoto, Katsura Matsumoto, Takeshi Kambara, Motoharu Hirano, Shoichi Maruyama, Noritoshi Kato, Yoshitaka Tatematsu, Shigeharu Ueki, Tomonori Nakazato

**Affiliations:** 1 Department of Hematology, Yokohama Municipal Citizen's Hospital, Yokohama, Japan; 2 Division of Transfusion and Cell Therapy, Tokyo Metropolitan Komagome Hospital, Tokyo, Japan; 3 Department of General Internal Medicine and Clinical Laboratory Medicine, Akita University Graduate School of Medicine, Akita, Japan; 4 Department of Infectious Disease, Yokohama Municipal Citizen's Hospital, Yokohama, Japan; 5 Department of Diagnostic Pathology, Yokohama City University Hospital, Yokohama, Japan; 6 Department of Pathology, Yokohama Municipal Citizen's Hospital, Yokohama, Japan; 7 Department of Dermatology, Keio University School of Medicine, Tokyo, Japan; 8 Department of Anesthesiology, Yokohama Municipal Citizen's Hospital, Yokohama, Japan; 9 Department of Nephrology, Yokohama Municipal Citizen's Hospital, Yokohama, Japan; 10 Department of Neurology, Yokohama Municipal Citizen's Hospital, Yokohama, Japan; 11 Department of Dermatology, Yokohama Municipal Citizen's Hospital, Yokohama, Japan; 12 Department of Rheumatology, Yokohama Municipal Citizen's Hospital, Yokohama, Japan; 13 Department of Nephrology, Nagoya University Graduate School of Medicine, Nagoya, Japan; 14 Department of Nephrology, Fujita Health University Bantane Hospital, Nagoya, Japan

## Introduction

Thrombotic microangiopathy (TMA) is a severe condition characterized by hemolytic anemia, thrombocytopenia, and renal impairment.^1^ Its etiology includes reduced activity of ADAMTS13, complementopathies, Shiga toxins, and a range of systemic conditions collectively categorized as “secondary TMA.” Among these, a few reports have described eosinophilia-associated TMA (EA-TMA).S1–S8 Nonetheless, the pathological role of eosinophils in TMA remains poorly understood. We present a case of EA-TMA in a young woman, marked by unprecedented severity and a fatal outcome. This case uniquely demonstrates the presence of Charcot-Leyden crystals (CLCs) in the kidney and offers insights into the contribution of eosinophils to TMA.

## Case Presentation

**Figure 1:**
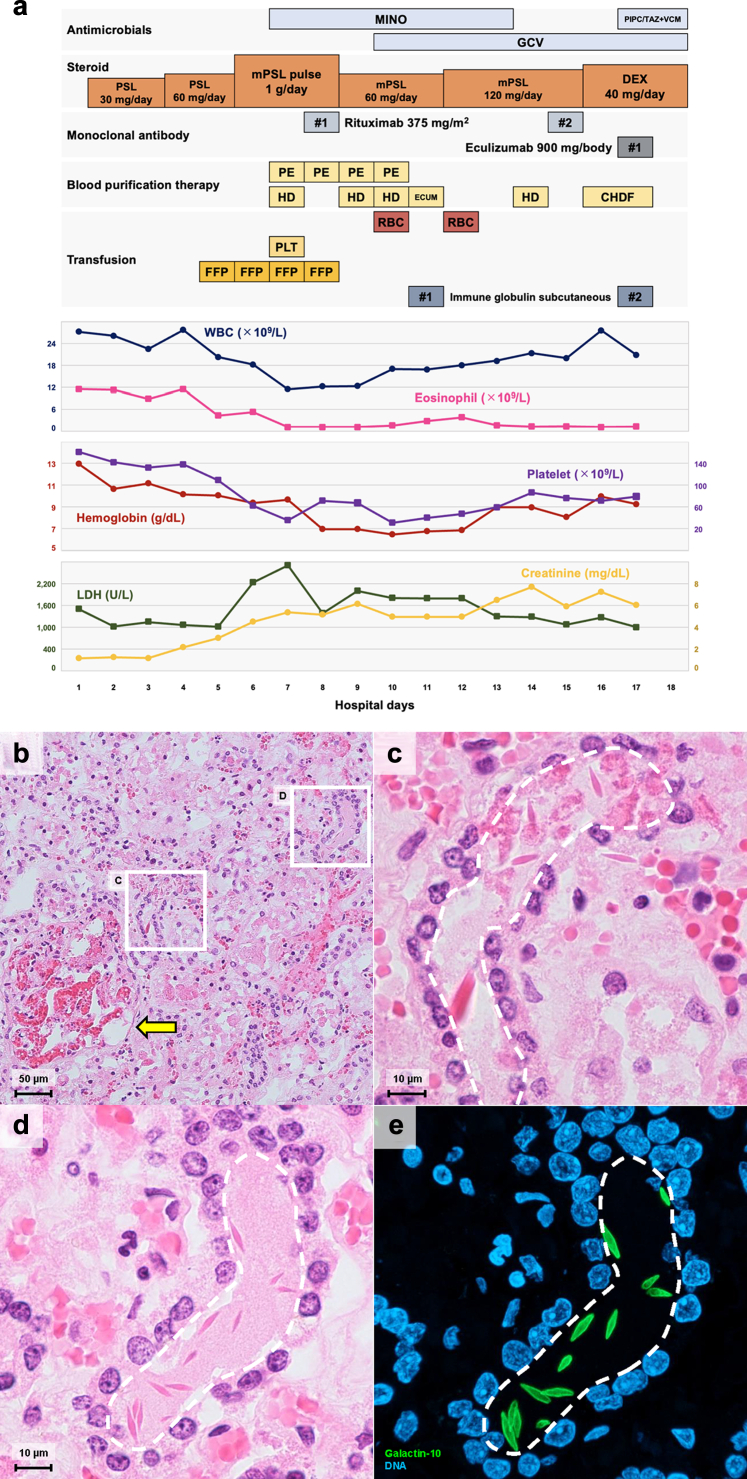
Clinical course and histological analysis. (a) Clinical course of the present case. The timeline illustrates the patient's clinical course after admission, including laboratory values and treatments administered. LDH, creatinine, white blood cell count, eosinophil count, hemoglobin, and platelet count are plotted against hospitalization days. Antimicrobial therapy, steroid therapy, monoclonal antibody administration, blood purification therapy, and transfusions are indicated accordingly. (b–e) Histological analysis of the kidney. The boxed areas in panels b (× 200) are magnified in panels C and D (× 1000). (b) Hematoxylin and eosin staining revealed glomerular thrombi (arrow) and needle-like crystals (boxed areas) were observed, but no eosinophils. (c and d) Crystals were observed mainly within the intratubular lumen (dotted areas) and some in the interstitium. (e) Identical sections are stained with anti–galectin-10 antibody (green) and Hoechst 33342 (blue) and assessed C region using confocal microscopy (ZEISS LSM980; Carl Zeiss, Jena, Germany). Z-stack images were acquired and reconstructed. Needle-shaped crystals were positive for galectin-10 and were confirmed to be Charcot-Leyden crystals. Immunostaining has been performed as previously described.^2^ CHDF, continuous hemodiafiltration; DEX, dexamethasone; ECUM, extracorporeal ultrafiltration membrane; FFP, fresh frozen plasma; GCV, ganciclovir; HD, hemodialysis; LDH, lactate dehydrogenase; MINO, minocycline; mPSL, methylprednisolone; PE, plasma exchange; PIPC/TAZ, piperacillin/tazobactam; PLT, platelet; PSL, prednisolone; RBC, red blood cell; VCM, vancomycin; WBC, white blood cell.

A 31-year-old female presented with fever and generalized erythema. She had no significant medical or allergy history. An allergic eruption was initially suspected, and she was treated with topical medications. However, she subsequently erythema developed diarrhea and edema, which necessitated hospitalization 10 days later. The day of admission was defined as day 1, and her clinical course after hospitalization is shown in Figure 1a.^2^

Blood tests revealed eosinophilia (white blood cells: 24.6 × 10^9^ /L, eosinophils: 39.5%). Bone marrow examination showed a similar eosinophil elevation (30.9%) without blast proliferation or morphological dysplasia. Chromosomal and genetic analyses of the bone marrow revealed no abnormalities. Positron emission tomography–computed tomography showed no abnormal uptake and colonoscopy revealed no evidence of malignancy. Idiopathic hypereosinophilic syndrome was suspected, and prednisolone 1 mg/kg was initiated on day 2 postadmission, which was then increased to 2 mg/kg on day 3. On the same day, she developed visual impairments, and by day 5, she exhibited a consciousness disorder (Glasgow coma scale: E4V4M6).

Cerebrospinal fluid examination and head magnetic resonance imaging showed no abnormalities. On day 6, hemolytic anemia (hemoglobin: 9.4 g/dl, schistocytes: 1%, lactate dehydrogenase: 2187 U/l; and undetectable haptoglobin), thrombocytopenia (63 × 10^9^ /L), and renal impairment (blood urea nitrogen: 61.5 mg/dl, creatinine: 4.41 mg/dl) led to a diagnosis of TMA. She was transferred to the intensive care unit and steroid pulse therapy (methylprednisolone: 1 g for 3 days) was commenced. Hemodialysis was initiated on day 8, along with 4 daily sessions of plasma exchange. Given the suspicion of thrombotic thrombocytopenic purpura (TTP) or atypical hemolytic uremic syndrome, rituximab (375 mg/m^2^) was administered on days 9 and 16, followed by eculizumab (900 mg/body) on day 17. Despite these treatments, anuria and impaired consciousness persisted. Subsequently, she developed seizures and cerebral edema and died on day 18.

We considered multiple etiologies for TMA, including Shiga toxin–associated hemolytic uremic syndrome; TTP; atypical hemolytic uremic syndrome; and secondary causes associated with infections, autoimmune diseases, malignancies, transplantation, pregnancy, or medications (Supplementary Table S1).S9–S11 Laboratory tests showed a negative anti–*Escherichia coli* O157 IgM, normal ADAMTS13 activity without inhibitors, a negative sheep red blood cell hemolysis test, anti–H factor antibodies, and no pathogenic variants in complement-related genes. Extensive infectious, autoimmune, and antiphospholipid antibody evaluations were negative (Supplementary Table S2). In the absence of an alternative cause, eosinophilia was deemed to be the primary driver, supporting diagnosis of EA-TMA.

At autopsy, the kidneys exhibited extensive glomerular thrombi (Figure 1b). Bone marrow eosinophils had returned to the normal range. Although the kidneys showed no significant eosinophil infiltration, needle-like crystals were observed in the interstitium and intratubular lumen (Figure 1c and d). These crystals were morphologically consistent with CLCs. Immunofluorescence and hematoxylin–eosin staining confirmed galectin-10 positivity, validating their identity as CLCs (Figure 1e).^2^ The brain showed significant edema, but no malignant findings or demyelination were observed. No malignant findings were observed in any other organs.

## Discussion

CLCs are characterized by their elongated, hexagonal bipyramidal shape connected at their bases.^3^ They are a classical pathological feature of eosinophilic inflammation. In this case, although routine staining did not reveal eosinophil infiltration in the kidneys, numerous CLCs were observed. CLCs are formed through the crystallization of galectin-10 during active eosinophil cytolysis.^2^ Recent studies have shown that CLCs can independently trigger inflammation and immune responses,S12 and are now recognized among the crystal-induced diseases, or “crystallopathies.”^4^ Although CLCs in the kidney have been reported in eosinophilic granulomatosis with polyangiitis,^5^ this is the first report of their presence in EA-TMA. In this case, the observed reduction of eosinophils was likely from steroid therapy; nonetheless, the residual CLCs suggest that intense eosinophilic inflammation may have contributed to the renal damage.

Eosinophilia is thought to increase thrombotic risk through several mechanisms.^6^ Activated eosinophils release granule proteins, such as major basic protein, which can directly induce endothelial cell injury. These proteins may also promote thrombosis by modulating the coagulation system by activating platelets and impairing the anticoagulant activity of thrombomodulin.S13^,^S14 In eosinophilic granulomatosis with polyangiitis, the concept of ”immunothrombosis” has been proposed, involving eosinophil extracellular traps formation via cytolysis, leading to platelet aggregation and activation.^7^ Such excessive eosinophilic inflammation may contribute to the pathogenesis of EA-TMA.

Previously reported cases of EA-TMA are summarized in Supplementary Table S3.S1–S8 Nine cases have been identified, with a median patient age of 31 years (range: 15–80 years); 7 patients were males and 2 were females. Eosinophilia was idiopathic in 7 cases and neoplastic (FIP1L1/PDGFRα-positive) in 2. Reported TMA subtypes included TTP in 4 cases, atypical hemolytic uremic syndrome in 1 case, and other TMA in 3 cases. Eight of 9 patients received corticosteroids, and additional treatments included imatinib for neoplastic cases, rituximab, plasma exchange, hemodialysis, and immunoglobulin therapy. In our case, we evaluated for neoplastic, allergic, drug-induced, parasitic, and autoimmune causes, but similar to TMA workup, no underlying cause was identified (Supplementary Table S4).S15–S16

In Table 1, we outline the principal clinical insights. The present case serves as a stark reminder that EA-TMA can be fatal even in young individuals. In 1924, Dr. Eli Moschcowitz described the sudden onset of petechiae and pallor, rapidly followed by paralysis, coma, and death in a 16-year-old girl, thereby establishing the concept of TTP.^8^ This historical milestone laid the foundation for the broader understanding of TMA, including secondary forms. Although the present case did not fulfill the criteria for TTP, it highlights the importance of recognizing atypical presentations of TMA associated with eosinophilia. In recent years, improvement in both eosinophilia and TMA has been reported with the anti-IL-5 antibody mepolizumab, suggesting potential therapeutic benefit.S6 Continued efforts to define disease and develop targeted treatments are urgently needed.

**Table 1:** Teaching points

• EA-TMA is a rare but life-threatening subtype of thrombotic microangiopathy, requiring early recognition and exclusion of other causes.
• Tissue Charcot–Leyden crystals serve as pathological evidence of previous eosinophil activation and may indicate chronic disease activity.
• Targeting eosinophilic inflammation may represent a rational therapeutic approach in the management of EA-TMA.

EA-TMA, eosinophilia-associated thrombotic microangiopathy.

### Conclusion

This case highlights the potential role of CLCs in EA-TMA, representing the first report of CLCs in this context. Given their emerging recognition as proinflammatory mediators, further research is essential to elucidate their contribution to endothelial injury and thrombotic processes in EA-TMA.

## Disclosure

SU received honoraria from AstraZeneca, GlaxoSmithKline, and Sanofi and grants from AstraZeneca, VIB, Cytrill, and Maruho Co. Ltd. All the other authors declared no competing interests.

## Patient Consent

The authors declared that they have obtained consent from the patient discussed in the report.

## Supplementary Material

Supplementary File (PDF) **Supplementary References. Table S1.** Differential diagnosis of thrombotic microangiopathy in the present case. **Table S2.** Results of infectious disease testing in the present case. **Table S3.** Review of published cases with eosinophilia-associated thrombotic microangiopathy. **Table S4.** Differential diagnosis of eosinophilic disorders in the present case.

## Footnotes

Supplementary File (PDF)

**Supplementary References.**
**Table S1.** Differential diagnosis of thrombotic microangiopathy in the present case. **Table S2.** Results of infectious disease testing in the present case. **Table S3.** Review of published cases with eosinophilia-associated thrombotic microangiopathy. **Table S4.** Differential diagnosis of eosinophilic disorders in the present case.
