# Supplementary material for: Fatal Eosinophilia-Associated Thrombotic Microangiopathy and the Role of Renal Charcot-Leyden Crystals
Source: Kidney Int Rep. 2025 May 19;10(8):2864–8. doi: 10.1016/j.ekir.2025.05.015 (PMC12347802; doi:10.1016/j.ekir.2025.05.015)
Supplement: Supplementary File (PDF) — Supplementary References. Table S1. Differential diagnosis of thrombotic microangiopathy in the present case. Table S2. Results of infectious disease testing in the present case. Table S3. Review of published cases with eosinophilia-associated thrombotic microangiopathy. Table S4. Differential diagnosis of eosinophilic disorders in the present case. [file mmc1.pdf]

Supplementary materials

Table S1. Differential diagnosis of thrombotic microangiopathy in the present case 2

Table S2. Results of infectious disease testing in the present case 3

Table S3. Review of published cases with eosinophilia-associated thrombotic microangiopathy 4

Table S4. Differential diagnosis of eosinophilic disorders in the present case 5

Supplementary References 6

Table S1. Differential diagnosis of thrombotic microangiopathy in the present case

**Definition of TMA**	**Examination**		**Results**	**Normal range**
Hemolytic anemia	Hemoglobin		7.0 g/dL	12.1–14.5 g/dL
	Reticulocyte		2.7%	0.4–1.9%
	Schistocytes		1.0%	
	Total bilirubin		1.5 mg/dL	0.2–1.2mg/dL
	Lactate dehydrogenase		2,611 U/L	120–245 U/L
	Haptoglobin		<=10 mg/dL	83–209 mg/dL
	Direct antiglobulin test		Negative	
Thrombocytopenia	Platelet		36×10^{9} /L	$150–350\times10^{9}$ /L
Endo-organ ischemia	Autopsy	Brain	Not detected	
		Heart	Not detected	
		Lung	Not detected	
		Gastrointestinal tract	Not detected	
		Kidney	Glomerular thrombi	
**Differential diagnosis**	**Examination**		**Results**	**Normal range**
STEC-HUS	Stool culture		Negative	
	Anti-LPS antibodies	IgM	Negative	
Acquired TTP **[S9]**	ADAMTS13 activity		63%	>=10%
	ADAMTS13 inhibitor		<0.5 BU/mL	<0.5 BU/mL
Atypical HUS	Sheep erythrocyte hemolytic assay **[S10]**		Negative	
	Anti-factor H antibodies		Negative	
	Mutations in complement pathway **[S11]**	*CFH*	Not detected	
		CFI	Not detected	
		CFB	Not detected	
		C3	Not detected	
		CD46	Not detected	
		THBD	Not detected	
		DGKE	Not detected	
Secondary TMA				
1. Infection	**Refer to Supplementary Table 2**			
2. Autoimmune diseases	Antinuclear antibody		<40 times	<40 times
	Anti-Scl-70 antibody		Negative	
	Anti-centromere antibody		<10 U/mL	<10 U/mL
	MPO-ANCA		<3.5 U/mL	<3.5 U/mL
	PR3-ANCA		<3.5 U/mL	<3.5 U/mL
	Lupus anticoagulant (dRVVT)		0.9	<1.2
	Lupus anticoagulant (APTT)		0.67	<1.16
	Anticardiolipin antibody	IgG	<=4.0 U/mL	<12.3 U/mL
	Anti–β2-glycoprotein I antibody	IgG	<=1.2 U/mL	<3.5 U/mL
3. Malignancies	Histopathology	Bone marrow	No evidence of malignancy	
		Skin	No evidence of malignancy	
		Colon	No evidence of malignancy	
	Cytopathology	Cerebrospinal fluid	No evidence of malignancy	
		Pleural effusion	No evidence of malignancy	
	Autopsy		No evidence of malignancy	
4. Transplantation			No history	
5. Pregnancy			No history	
6. Drug			No history	

ADAMTS13, a disintegrin and metalloproteinase with thrombospondin motifs 13; APTT, activated partial thromboplastin time; BU, Bethesda unit; C3, complement component 3; CFB, complement factor B; CFH, complement factor H; CFI, complement factor I; dRVVT, dilute Russell viper venom time; DGKE, diacylglycerol kinase epsilon; HUS, hemolytic uremic syndrome; LPS, lipopolysaccharide; MPO-ANCA, myeloperoxidase-antineutrophil cytoplasmic antibody; PR3-ANCA, proteinase 3-antineutrophil cytoplasmic antibody; STEC-HUS, Shiga toxin-producing Escherichia coli-hemolytic uremic syndrome; THBD, thrombomodulin; TMA, thrombotic microangiopathy; TTP, thrombotic thrombocytopenic purpura.

Table S2. Results of infectious disease testing in the present case

Method	Pathogen	Specimen	Hospital day	Results
Culture		Blood	Day 1	Negative
			Day 6	Negative
			Day 12	Negative
			Day 17	Negative
		Cerebrospinal fluid	Day 6	Negative
			Day 16	Negative
		Pleural effusion	Day 15	Negative
Antigen	*Streptococcus pneumoniae*	Cerebrospinal fluid	Day 6	Negative
	Legionella	Urine	Day 7	Negative
Antibody	*Orientia tsutsugamushi*	Blood	Day 7	Karp IgG: Negative
				Karp IgM: Negative
				Gilliam IgG: Negative
				Gilliam IgM: Negative
				Kato IgG: Negative
				Kato IgM: Negative
	Dirofilaria immitis	Blood	Day 7	Equivocal
	Toxocara canis			Negative
	Ascaris suum			Negative
	Anisakis			Negative
	Trichuris trichiura			Negative
	Ascaris lumbricoides			Negative
	Paragonimus westermani			Negative
	Paragonimus miyazakii			Negative
	Clonorchis sinensis			Negative
	Fasciola hepatica			Negative
	Taenia solium			Negative
	Echinococcus granulosus			Negative
Virus culture in Vero cells		Throat swab	Autopsy	Negative
		Cerebrospinal fluid		Negative
Qualitative PCR	Adenovirus	Cerebrospinal fluid	Autopsy	Negative
	Parvovirus B19			Negative
	Enterovirus/Rhinovirus			Negative
	Herpes simplex virus (HSV)			Negative
	Human parechovirus (HPeV)			Negative
	Parainfluenza virus			Negative
	Varicella zoster virus (VZV)			Negative
Real-time PCR	Human herpes virus 6 (HHV-6)	Blood	Day 7	1.3×10^{2} copies/10^{6} cells
		Cerebrospinal fluid	Day 7	Below detection limit
			Day 16	Below detection limit
	Cytomegalovirus (CMV)	Blood	Day 14	Below detection limit
	Epstein-Barr Virus	Blood	Day 7	Below detection limit
		Cerebrospinal fluid	Day 16	Below detection limit
Real-time PCR	Adenovirus	Throat swab	Autopsy	Below detection limit
	Enterovirus			Below detection limit
	Human bocavirus (HBoV)			Below detection limit
	Human coronavirus 229E			Below detection limit
	Human coronavirus HKU1			Below detection limit
	Human coronavirus NL63			Below detection limit
	Human coronavirus OC43			Below detection limit
	Human metapneumovirus (HMPV)			Below detection limit
	Human parechovirus (HPeV)			Below detection limit
	Influenza A H1pdm			Below detection limit
	Influenza A H3			Below detection limit
	Influenza B			Below detection limit
	Mycoplasma pneumoniae			Below detection limit
	Parainfluenza virus 1			Below detection limit
	Parainfluenza virus 2			Below detection limit
	Parainfluenza virus 3			Below detection limit
	Parainfluenza virus 4			Below detection limit
	Parvovirus B19			Below detection limit
	Respiratory syncytial virus (RSV)			Below detection limit
	Rhinovirus			Below detection limit

Table S3. Review of published cases with eosinophilia-associated thrombotic microangiopathy

Year [Reference]	Age [y.o.]	Sex	Cause of eosinophilia	Cause of TMA	Medications	Renal replacement therapy	Observation period	Outcome
2005 **[S1]**	22	Female	Idiopathic	TTP	Imatinib	Plasma exchange<br>Hemodialysis	16 days	Survived
2005 **[S2]**	26	Male	Idiopathic	Not described	Steroid<br>Imatinib	None	12 months	Survived
2005 **[S2]**	15	Male	Idiopathic	Not described	Steroid<br>Rituximab<br>Immunoglobulin<br>Cyclophosphamide	Plasma exchange	6 months	Survived
2009 **[S3]**	80	Female	Idiopathic	TTP	Steroid	Plasma exchange	6 months	Survived
2012 **[S4]**	49	Male	Eosinophilic pneumonia	Other TMA	Steroid	Plasma exchange	6 months	Survived
2013 **[S5]**	24	Male	Neoplastic<br>(*FIP1L1-PDGFRA* fusion)	Other TMA	Imatinib	None	12 months	Survived
2018 **[S6]**	31	Male	Idiopathic	TTP/aHUS	Steroid<br>Montelukast<br>Mepolizumab	None	Not described	Survived
2019 **[S7]**	36	Male	Neoplastic<br>(*FIP1L1-PDGFRA* fusion)	Suspected TTP	Steroid, Imatinib	Plasma exchange	28 months	Survived
2019 **[S8]**	63	Male	Idiopathic	Other TMA	Steroid, Heparin, Warfarin	None	12 months	Survived

aHUS, atypical hemolytic uremic syndrome; TMA, thrombotic microangiopathy; TTP, thrombotic thrombocytopenic purpura; y.o., years old.

Table S4. Differential diagnosis of eosinophilic disorders in the present case

Differential diagnosis [S15, S16]	The present case
**Secondary causes**	
Allergy and hypersensitivity	Undetectable
Drug reactions	Undetectable
Parasitic infection	Undetectable
Collagen vascular disease	Undetectable
Inflammatory bowel disease	Undetectable
Solid tumor/ lymphoma	Undetectable
Metabolic condition (e.g. adrenal insufficiency)	Undetectable
**Myeloid/lymphoid neoplasms associated with eosinophilia and rearrangements of *PDGFRA*, *PDGFRB*, or *FGFR1*, or with *PCM1-JAK2***	
Chromosomal karyotype	Normal karyotype
FISH: *FIP1L1-PDGFRA* fusion gene	0%
FISH: *ETV6-PDGFRB* fusion gene	0%
FISH: 8p11 translocation	0%
**Chronic eosinophilic leukemia-not otherwise specified (CEL-NOS)**	
There is eosinophilia (eosinophil count > 1.5×10^9/L)	Yes
Not meeting WHO criteria for BCR-ABL1-positive chronic myeloid leukemia, PV, ET, PMF, CNL, CMML, or atypical CML	Yes
No rearrangement of *PDGFRA*, *PDGFRB*, or *FGFR1*; no *PCM1-JAK2*, *ETV6-JAK2*, or BCR-JAK2 fusion gene	Yes
The blast cell count in the peripheral blood and BM is less than 20%, and inv(16)(p13.1q22), t(16;16)(p13;q22), and other diagnostic features of AML are absent	Yes
There is a clonal cytogenetic or molecular genetic abnormality, or blast cells are ≥2% in the peripheral blood or >5% in the BM	No
**Lymphocytic variant of hypereosinophilic syndrome (L-HES)**	
Abnormal T-cell immunophenotype (and clonal T-cell receptor gene rearrangement)	Undetectable

Supplementary References

S1. Al Aly Z, Philoctete AJM, Gellens ME, Gonzalez EA. Thrombotic thrombocytopenic purpura in a patient treated with imatinib mesylate: true association or mere coincidence? *Am J Kidney Dis*. 2005;45:762-768. http://dx.doi.org/10.1053/j.ajkd.2004.12.017

S2. Liapis H, Ho AK, Brown D, Mindel G, Gleich G. Thrombotic microangiopathy associated with the hypereosinophilic syndrome. *Kidney Int*. 2005;67:1806-1811. http://dx.doi.org/10.1111/j.1523-1755.2005.00278.x

S3. Ohguchi H, Sugawara T, Harigae H. Thrombotic thrombocytopenic purpura complicated with hypereosinophilic syndrome. *Intern Med*. 2009;48:1687-1690. http://dx.doi.org/10.2169/internalmedicine.48.2282

S4. Yuste C, Quiroga B, Verde E, et al. The non-casual relation between eosinophilia and thrombotic microangiopathy. *Transfus Apher Sci*. 2012;47:365-367. http://dx.doi.org/10.1016/j.transci.2012.07.021

S5. Langlois AL, Shehwaro N, Rondet C, et al. Renal thrombotic microangiopathy and FIP1L1/PDGFRalpha-associated myeloproliferative variant of hypereosinophilic syndrome. *Clin Kidney J*. 2013;6:418-420. http://dx.doi.org/10.1093/ckj/sft067

S6. Mulvey JJ, Magro C, Chadburn A. Resolution of a steroid-resistant, hypereosinophilic immune diathesis with mepolizumab and concomitant amelioration of a mixed thrombotic microangiopathy. *Blood Cells Mol Dis*. 2018;69:38-42.

http://dx.doi.org/10.1016/j.bcmd.2017.04.008

S7. Alshehri H, Alnomani M, Alghamdi M, et al. An intriguing case of eosinophilia with FIP1L1/PDGFRA rearrangement who presented as thrombotic thrombocytopenic purpura. *Case Rep Hematol*. 2019;2019:2820954. http://dx.doi.org/10.1155/2019/2820954

S8. Curras-Martin D, Patel S, Qaisar H, et al. Acute kidney injury secondary to thrombotic microangiopathy associated with idiopathic hypereosinophilic syndrome: a case report and review of the literature. *J Med Case Rep*. 2019;13:281. http://dx.doi.org/10.1186/s13256-019-2187-4

S9. Matsumoto M, Miyakawa Y, Kokame K, et al. Diagnostic and treatment guidelines for thrombotic thrombocytopenic purpura (TTP) in Japan 2023. *Int J Hematol*. 2023;118:529-546. http://dx.doi.org/10.1007/s12185-023-03657-0

S10. Yoshida Y, Miyata T, Matsumoto M, et al. A novel quantitative hemolytic assay coupled with restriction fragment length polymorphisms analysis enabled early diagnosis of atypical hemolytic uremic syndrome and identified unique predisposing mutations in Japan. PLoS One. 2015;10:e0124655. http://dx.doi.org/10.1371/journal.pone.0124655

S11. Yoshida Y, Kato H, Ikeda Y, et al. Pathogenesis of Atypical Hemolytic Uremic Syndrome. J Atheroscler Thromb. 2019;26:99-110. http://dx.doi.org/10.5551/jat.RV17026

S12. Persson EK, Verstraete K, Heyndrickx I, et al. Protein crystallization promotes type 2 immunity and is reversible by antibody treatment. *Science*. 2019;364(6442).

http://dx.doi.org/10.1126/science.aaw4295

S13. Rohrbach MS, Wheatley CL, Slifman NR, Gleich GJ. Activation of platelets by eosinophil granule proteins. *J Exp Med*. 1990;172:1271-1274. http://dx.doi.org/10.1084/jem.172.4.1271

S14. Slungaard A, Vercellotti GM, Tran T, Gleich GJ, Key NS. Eosinophil cationic granule proteins impair thrombomodulin function. A potential mechanism for thromboembolism in hypereosinophilic heart disease. *J Clin Invest*. 1993;91:1721-1730. http://dx.doi.org/10.1172/jci116382

S15. Valent P, Klion AD, Roufosse F, et al. Proposed refined diagnostic criteria and classification of eosinophil disorders and related syndromes. *Allergy*. 2023;78:47-59. http://dx.doi.org/10.1111/all.15544

S16. Mattis DM, Wang SA, Lu CM. Contemporary Classification and Diagnostic Evaluation of Hypereosinophilia. *Am J Clin Pathol*. 2020;154:305-318. http://dx.doi.org/10.1093/ajcp/aqaa056
